# Optimizing irrigation and nitrogen levels for improved soil nitrogen dynamics and use efficiency in temperate ecology of Kashmir

**DOI:** 10.1038/s41598-025-32465-1

**Published:** 2025-12-30

**Authors:** Mohd Salim Mir, Waseem Raja, Raihana Habib Kanth, Eajaz Ahmad Dar, Zahoor Ahmad Shah, Danishta Aziz, Laila A. Al-Shuraym, Lamya Ahmed Alkeridis, Parmeet Singh, Amal Saxena, Lal Singh, Umer Fayaz, Satyapriya, Amged El-Harairy, Ahmed A. A. Aioub, Samy Sayed

**Affiliations:** 1 Division of Agronomy, Faculty of Agriculture, Sher-E-Kashmir University of Agricultural Sciences and Technology of Kashmir, WaduraSopore, 193201 India; 2 Sher-E-Kashmir University of Agricultural Sciences and Technology of Kashmir, Wadura, 190006 India; 3 West Florida Research and Education Centre, University of Florida, Jay, FL 32565 USA; 4 Division of Agricultural Extension, Faculty of Agriculture, Sher-E-Kashmir University of Agricultural Sciences and Technology of Kashmir, WaduraSopore, 193201 India; 5 Division of Entomology, Faculty of Agriculture, Sher-E-Kashmir University of Agricultural Sciences and Technology of Kashmir, WaduraSopore, 193201 India; 6 Department of Biology, College of Science, Princess Nourah Bint Abdulrahman University, P. O. Box 84428, 11671 Riyadh, Saudi Arabia; 7 Directorate of Research, Sher-E-Kashmir University of Agricultural Sciences and Technology of Kashmir, WaduraSopore, 190025 India; 8 Division of Genetics and Plant Breeding, Faculty of Agriculture, Sher-E-Kashmir University of Agricultural Sciences and Technology of Kashmir, WaduraSopore, 193201 India; 9 Head and Principal Scientist (Agril Extension), Division of Agricultural Extension, ICAR-IARI, New Delhi, India; 10 Department of Crop and Animal Sciences, Albrecht Daniel Thaer-Institute of Agricultural and Horticultural Sciences, Faculty of Life Sciences, Humboldt-Universität Zu Berlin, Albrecht-Thaer-Weg 5, 14195 Berlin, Germany; 11 Unit of Entomology, Plant Protection Department, Desert Research Center, 1 Mathaf El-Matariya St., El-Matariya, Cairo, 11753 Egypt; 12 Department of Plant Protection, Faculty of Agriculture, Zagazig University, Zagazig, 44511 Sharkia Egypt; 13 Department of Economic Entomology and Pesticides, Faculty of Agriculture, Cairo University, Giza, 12613 Egypt

**Keywords:** Irrigation, Nitrogen, Nutrient uptake, Transplanted rice, Flooded rice, Nitrogen use efficiency, Ecology, Ecology, Environmental sciences, Plant sciences

## Abstract

The present study aimed to evaluate nitrogen dynamics and use efficiency of transplanted rice under variable irrigation regimes and nitrogen levels. Two field experiments were conducted during the 2021 and 2022 rice-growing seasons using a split-plot design with four irrigation treatments in the main plots and four nitrogen levels in the sub-plots, each replicated thrice. Results indicated that nitrogen concentration and uptake by grain and straw were significantly influenced by both irrigation scheduling and nitrogen application. Among irrigation treatments, recommended scheduling and irrigation at field capacity produced the highest nitrogen concentration and uptake, whereas 10 and 20% depletion from field capacity resulted in lower values. For nitrogen levels, 125% of the recommended dose (RDN) recorded the highest grain nitrogen content and uptake, but values were statistically similar to 100% RDN. Flooded rice cultivation led to the greatest nitrogen removal from soil, followed by field capacity and deficit irrigation treatments. The highest nitrogen use efficiency was observed under deficit irrigation, followed by field capacity, while flooded irrigation was the least efficient. Adequate irrigation (I_1_/I_2_) resulted in the highest nitrogen uptake and grain yield, while deficit irrigation (I_4_) saved water but led to lower yield and nitrogen use efficiency, with greater amounts of residual nitrogen remaining in the soil. Overall, applying irrigation at field capacity combined with 100% RDN was found optimal for maximizing nutrient uptake and nitrogen use efficiency in transplanted rice, suggesting a sustainable approach to improve resource use without over-application of water or fertilizer.

**Supplementary Information:**

The online version contains supplementary material available at 10.1038/s41598-025-32465-1.

## Introduction

Rice is a staple food for nearly half of the world’s population, playing a key role in global food security^1,2^. It is cultivated in 118 countries^3^, producing 517.6 million tonnes^4^. In Asia, China leads with 148.3 million tonnes, followed by India with 120 million tonnes in 2020–21^5^. Irrigated rice is particularly important for regional food and livelihood security^6^. In India, rice covers 44.5 million hectares and contributes 40–43% (116.42 million tonnes) of total food grain production^7^. In Kashmir, rice is the primary staple food and a major component of local food security^8^. Water and soil are vital natural resources for agriculture, yet they are intangible and vulnerable to depletion^9^. Agriculture consumes about 85% of global freshwater^10^, with rice requiring 3000–5000 L of water per kg of grain^11^. Irrigated rice alone accounts for 40% of global irrigation water and nearly 80% of Asia’s irrigated freshwater^12^. Declining surface and groundwater resources threaten rice production^13,14^, necessitating strategies to improve water productivity^15^. Water-saving practices such as alternate wetting and drying^16^, aerobic rice^17^, direct-seeded rice^18^, continuous soil saturation^13^, the system of rice intensification^6^, and raised bed cultivation^19^ have proven effective. Implementing sustainable strategies, such as effective water management and soil fertility techniques, has demonstrated the ability to boost rice yields by up to 50% while reducing water consumption by 15%^20^. Although water-saving techniques such as alternate wetting and drying (AWD), aerobic rice, and the system of rice intensification (SRI) have been widely explored, they have limitations in temperate Himalayan conditions. AWD may delay crop growth under low temperatures, aerobic rice can reduce yield in cooler climates, and SRI demands intensive management and skilled labor. These constraints underscore the need for precision approaches like sensor-based irrigation, which allows real-time soil moisture monitoring and optimized water application, offering a more sustainable and efficient solution for rice cultivation in this region. The use of optimized and intelligent irrigation systems in rice cultivation has proven effective in conserving water without negatively impacting yields or other agronomic factors^21^. In India, smart sensor-based irrigation for transplanted rice has saved 41% water compared to conventional flooding^10^. Compared to the traditional method of continuous flooding, these optimized management practices can enhance water productivity by 34% and decrease water usage by 40%^22^. Optimized irrigation scheduling using smart irrigation systems relies on sensors to monitor plant, soil, and weather conditions^23^. Soil moisture monitoring, through measurements of soil water potential or content, is a widely adopted approach for efficient irrigation scheduling^24^. Understanding soil moisture dynamics and its interaction with irrigation and plant water uptake underscores the importance of monitoring within the root zone^25^. Deploying soil moisture sensors at multiple soil depths allows precise tracking of moisture variations, enhancing irrigation accuracy and improving insights into how soil water content responds to irrigation and crop water use^26^. Nitrogen is critical for rice growth, supporting nucleotides, amino acids, and chlorophyll synthesis^27^. Its deficiency limits biomass and grain yield^28^. Therefore, nitrogen is crucial for agriculture and global food security, and fertilizers are necessary to meet the growing food demand^29^. However, nitrogen use efficiency in irrigated rice is low due to losses such as volatilization, denitrification, runoff, and leaching^30^. Optimized nitrogen management enhances yield, reduces cost, and improves environmental outcomes^31^. Sensor-based irrigation research in rice remains limited in Northern India especially in North-Western Himalayas, with virtually no studies conducted under the temperate conditions of Kashmir. This knowledge gap constrains region-specific recommendations for sustainable water and nitrogen management in rice cultivation. Recognizing this gap, the present study introduces a novel approach by integrating sensor-based irrigation scheduling with variable nitrogen rates to assess their combined effects on rice performance in temperate agroecosystems. We hypothesized that sensor-guided deficit irrigation, when coupled with optimized nitrogen management, could enhance nutrient content, nutrient uptake, and nitrogen use efficiency (NUE) without compromising yield potential. This approach is expected to provide deeper insights into nitrogen dynamics under controlled and deficit irrigation regimes, thereby contributing to resource-efficient and climate-resilient rice production strategies.

## Material and methods

### Experimental site details

**Fig. 1:**
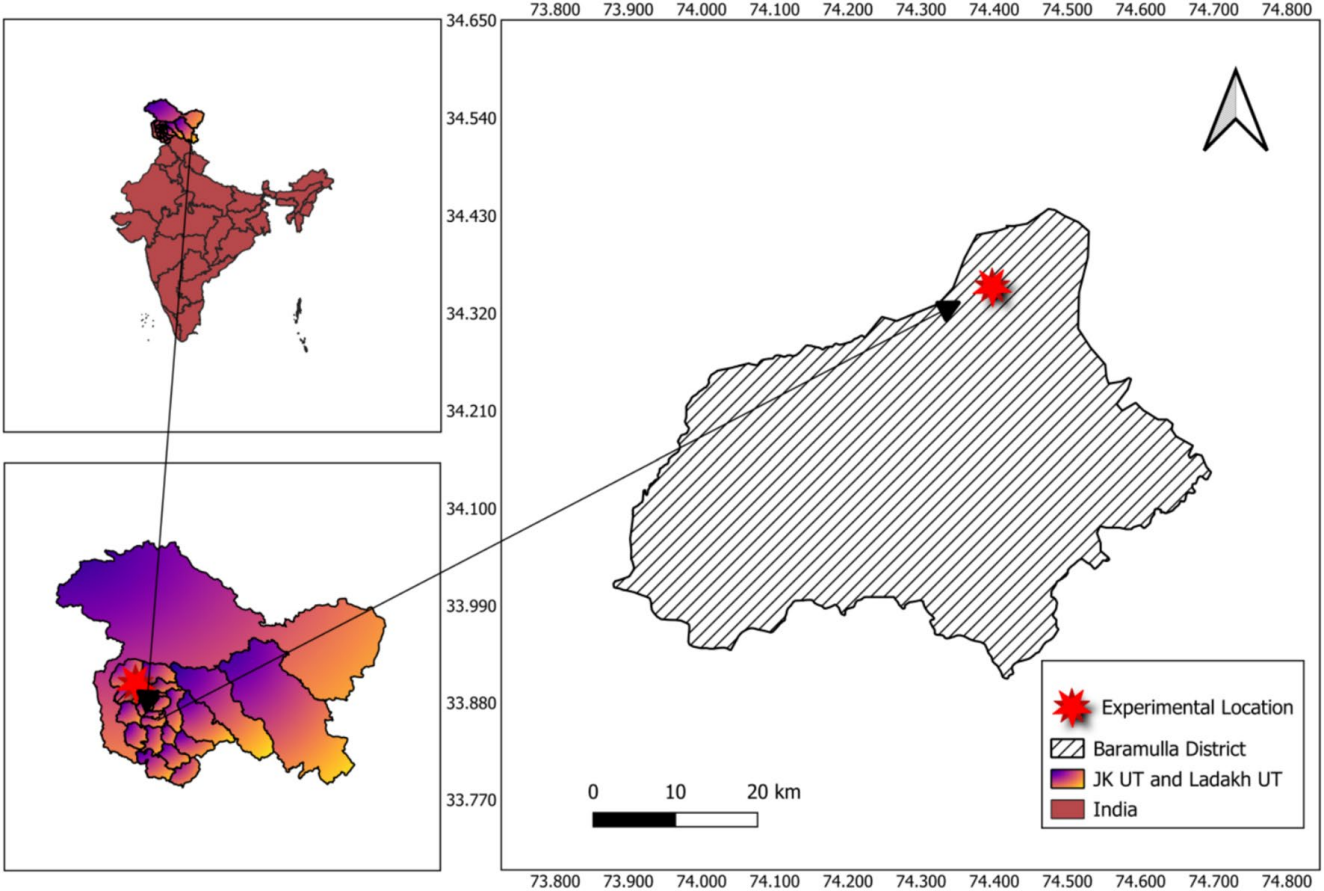
Location map of the experimental site.

The experiment was conducted at crop research farm of Division of Agronomy, Faculty of Agriculture, Sher-e-Kashmir University of Agricultural Sciences & Technology of Kashmir, Wadura, Sopore during *Kharif* 2021 and 2022. The experimental site is situated between latitude of 34°21′ N and longitude of 74°23′ E at an altitude of 1590 m above mean sea level (Fig. 1). Composite soil samples were collected from 0–10, 10–20, 20–30, 30–40, 40–60 and 60–100 cm soil depth from randomly selected spots before the layout of experiment, composited and subjected to mechanical and chemical analysis. The soil of the experimental site was silty clay loam in texture with sand, silt and clay content varying from 8.8–12.2%, 48.4–53.6% and 34.2–41.3% respectively (Table 1). The field capacity and saturated water content of different layers (0–10; 10–20; 20–30; 30–40; 40–60 and 60–100 cm) of soil calculated through field method varied from 0.295 to 0.421 m^3^ m^-3^ and 0.352 to 0.484 m^3^ m^-3^, respectively (Mir et al*.*^58^). The bulk density of the soil ranged between 1.32 and 1.37 Mg m^-3^ for different soil layers. Soil texture was determined by using International pipette method^32^. Bulk density and particle density of soil was determined through core sampler method and pycnometer^33^. Chemical analysis of the soil revealed that the soil was having normal electrical conductivity, medium in organic carbon, medium in available nitrogen, phosphorus and potassium with neutral pH (Table 2).

**Table 1:** Physical characteristics of the soil of the experimental field.

Depth (cm)	Field capacity (m^3^ m^-3^)	Bulk density (Mg m^-3^)	Saturated water content (m^3^ m^-3^)	Sand (%)	Silt (%)	Clay (%)
0–10	0.295	1.37	0.352	12.2	53.6	34.2
10–20	0.337	1.36	0.386	11.9	50.8	37.3
20–30	0.361	1.35	0.418	10.1	52.3	37.6
30–40	0.397	1.36	0.445	9.2	51.7	39.1
40–60	0.414	1.33	0.472	8.8	50.5	40.7
60–100	0.421	1.32	0.484	10.3	48.4	41.3

**Table 2:** Chemical characteristics of the soil of the experimental field.

Soil property	Value	Rating	Method employed
Electrical conductivity (dSm^-1^)	0.15	Normal	1:2.5 soil water suspension with Solubridge conductivity meter^34^
pH	6.9	Neutral	1:2.5 soil water suspension using Systronics pH meter^34^
Organic carbon (%)	0.88	Medium	Wet digestion method^35^
Available N (kg ha^-1^)	380	Medium	Modified alkaline permanganate method^36^
Available P_2_O_5_ (kg ha^-1^)	18.5	Medium	Extraction with 0.5 M NaHCO_3_^37^ using Systronics Spectrophotometer
Available K_2_O (kg ha^-1^)	272	Medium	Extraction with neutral normal NH_4_OAC using Systronics Flame Photometer^38^

### Climate and weather conditions

The climate is temperate cum Mediterranean and continental type characterized by hot summers and chilling winters. The average annual precipitation is 812 mm (average over past 30 years) and more than 80% of precipitation is received from western disturbances. The minimum and maximum temperature ranging between –8.0 to 33 °C, exhibits considerable fluctuation both in summer and winter.

**Fig. 2:**
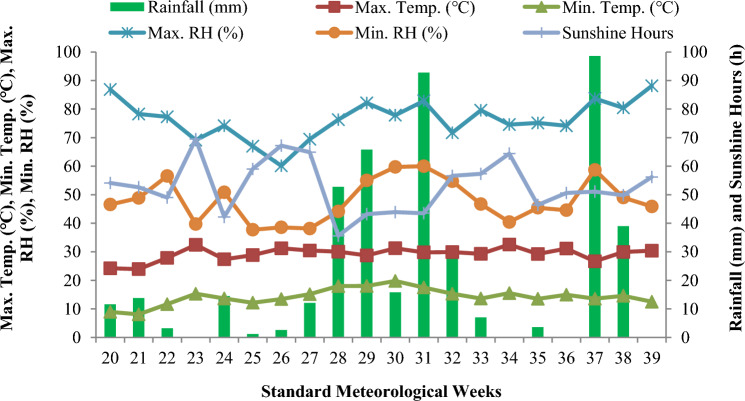
The temporal change in maximum and minimum temperature, maximum and minimum relative humidity, rainfall and sunshine hours during the 2021 growing season.

**Fig. 3:**
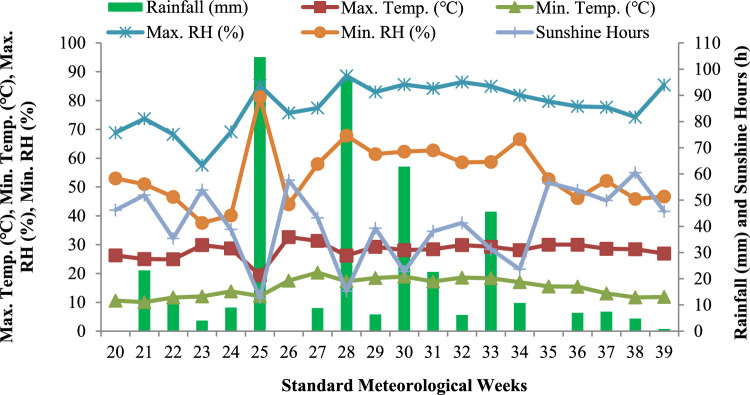
The temporal change in maximum and minimum temperature, maximum and minimum relative humidity, rainfall and sunshine hours during the 2022 growing season.

The mean meteorological data for the cropping seasons of 2021 and 2022 recorded at Meteorological Observatory at Division of Agronomy, Sher-e-Kashmir University of Agricultural Sciences and Technology of Kashmir, Shalimar is presented in Figs. 2 and 3. The minimum temperature ranged from 8.1 ℃ to 19.8 ℃ and 10.1 ℃ to 20.3 ℃, and maximum temperature ranged from 23.9 ℃ to 32.4 ℃ and 19.3 ℃ to 32.6 ℃ and the average maximum relative humidity ranged from 60 to 88% and 57% to 88%, whereas mean minimum relative humidity ranged from 38 to 60% and 37% to 81% during the crop growing seasons of 2021 and 2022, respectively. The average number of sunshine hours per standard meteorological week in 2021 and 2022 were 53 and 41, respectively. The total rainfall received during the experimentation period was 463 mm and 432 mm during 2021 and 2022 respectively.

### Experimental design and treatment details

**Fig. 4:**
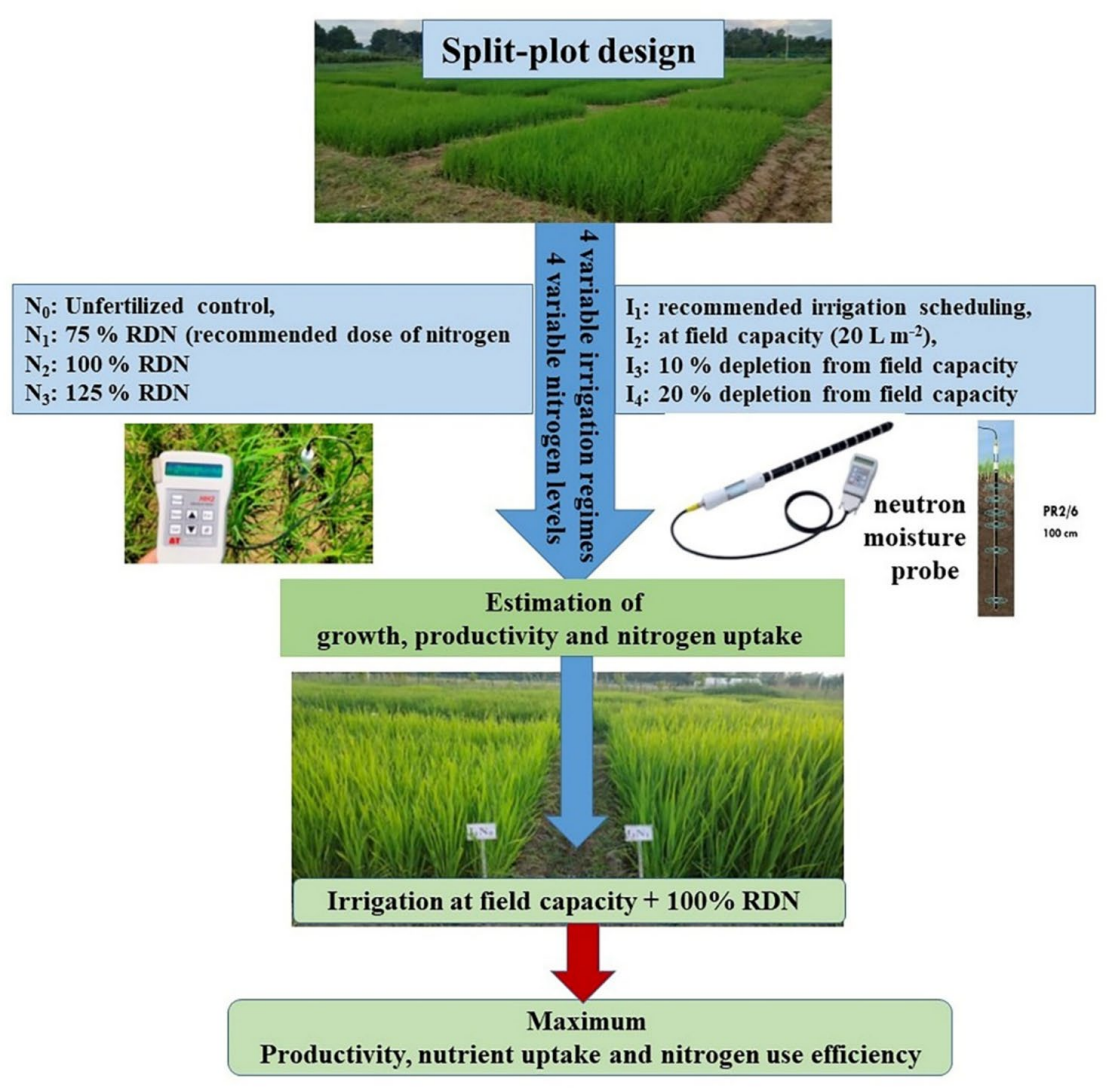
Experimental design and treatment details of irrigation and fertilization.

The experiment was laid out in split plot design with four main plot treatments viz. I_1_: recommended irrigation scheduling**,** I_2_: at field capacity (20 L m^-2^), I_3_:10% depletion from field capacity (20 L m^-2^) and I_4_: 20% depletion from field capacity (20 L m^-2^) and four sub plot treatments viz. N_0_: Unfertilized control, N_1_: 75% RDN (recommended dose of nitrogen; @120 kg ha^-1^), N_2_: 100% RDN and N_3_: 125% RDN (Fig. 4). One-meter buffer zone was kept between the plots to hinder the flow of water between them, and the blocks were additionally separated by a distance of 1 m. The size of each experimental plot was 5 m × 3 m and the bunds of 15 cm were made to prevent water loss from the plots.

### Crop management practices

Main field was ploughed with tractor-drawn disc plough after harvesting of previous crop. Subsequently, three ploughings were given with tiller to bring the soil to a fine tilth and levelling was done manually with traditional land leveller. Replication borders, plot-paths, irrigation and drainage channels were constructed manually. Bunds as per treatments were made in dry condition and then whole field was irrigated for puddling and giving final shape to the bunds. The nursery beds of 3 m width and 2 m length were prepared. Drainage channels were provided along the bed to drain out the excess water. On these nursery beds well decomposed farm yard manure (FYM@5 kg m^-2^) mixture was spread as well as recommended dose of N, P and K was applied. Seeds of variety Shalimar Rice-4 were soaked for 48 h and incubated in moist gunny cloth for another 48 h for sprouting. Shalimar Rice-4 is an early maturing and high yielding variety developed by Mountain Research Centre for Field Crops (MRCFC) Khudwani of SKUAST-K and was released in 2017. It is a cold tolerant indica variety having resistance to blast, erect plant type, light green basal leaf sheath, high test weight, high biomass yield and easy threshability. It is highly recommended for cultivation in irrigated low lands of the Kashmir valley (up to 1700 m amsl). Its yield potential is 8.5–9.0 t ha^-1^ and matures in 135–140 days. Seed rate @ 60 kg ha^-1^ was used and seeds were broadcasted uniformly on the nursery beds. Water was allowed frequently into channels that were formed around the nursery beds. Seeds were treated with Tricyclazole 75 WP @ 0.6 g kg^-1^ seed to prevent seed rot and pre-emergence damping off & rice blast. The transplantation of 30 days old seedlings were done on June 17, 2021 and June 19, 2022 after proper puddling of plots with 4–5 seedlings per hill at a geometry of 15 cm × 15 cm between the hills. Well decomposed farmyard manure (FYM) @ 10 t ha^-1^ was applied to experimental sites at the time of lay out of the field. Full recommended dose of phosphorus and potassium through di-ammonium phosphate and muriate of potash at the rate of 60 and 30 kg P_2_O_5_ and K_2_O ha^-1^, respectively was uniformly applied to each plot as basal dose before transplanting. Nitrogen was applied as per treatments through urea with half as basal and remaining half in two equal splits i.e. tillering (18–22 DAT) and panicle initiation stages (35–40 DAT). Bensulfuron methyl + pretilachlor (60 g + 600 g a.i. ha^-1^) with the trade name of Erase @ 10 kg ha^-1^ was hand broadcasted 4 days after transplantation for weed control. It was followed by manual hand weeding after 15 days of herbicide application.

### Irrigation management

Installation of 7000-L capacity PVC tanks was carried out at a 2-m elevation, accompanied by the placement of a 1.5-inch water meter at the tank outlet. Preceding transplanting, a surface irrigation system was implemented, involving a PVC pipeline running parallel to the plots. Each plot featured a centrally located inlet equipped with a water-tight butterfly valve, guaranteeing the irrigation of one plot at a time within each replication. The pressure in the pipeline was maintained by installation of 1.5 hp pump installed at the outlet of the water tank behind the water meter. Another 2.0 hp pump was installed 20 feet away from the water source to feed the two PVC tanks. Irrigation water was administered for each treatment until the designated quantity was delivered. The measurement of volumetric soil moisture was conducted through the use of the Delta-T Devices PR2 soil moisture profile probe (Delta-T Device, UK). Access tubes were installed to a depth of 1 m in the soil for this purpose. Soil moisture levels were assessed at six different depths (0–10, 10–20, 20–30, 30–40, 40–60, and 60–100 cm) both prior to and following each irrigation application in all treatments. The amount of irrigation water administered to these treatments was recorded using a water meter installed in the main line. For the treatments based on depletion from field capacity, a management depth of 30 cm was considered. In treatment I2 to I4, irrigation depth was 20 mm and for I1, it was 50 mm per irrigation.

### Recommended irrigation scheduling

A 2–3 cm water level was maintained upto tillering stage. Water was drained out at mid-tillering stage, thereafter water level of 2–3 cm was maintained up to panicle initiation stage. Water was drained out from the field at panicle initiation stage and the crop was re-irrigated after hair like cracks appeared in the field. At pre-heading stage, again water was drained out to stimulate heading. From flowering to milk stage, a thin layer of water was maintained in the field. After semi-dough stage, alternate wetting and drying was followed up to physiological maturity.

### Nutrient studies

Plant samples collected at harvest were sun dried and then packed in labelled long paper bags. These samples were put in an electric oven, dried for 36 h at 60–65 ℃ temperature till constant weight was obtained. After recording the dry weight of plant samples (q ha^-1^) collected from each plot, oven dried samples of both grain and straw were ground in Wileys mill and passed through 32 mesh sieve and subsequently used for chemical analysis.

### Nitrogen content and uptake by rice

Nitrogen content (%) was estimated by digesting 0.5 g sample with 10 ml concentrated sulphuric acid and digestion mixture. Total nitrogen was determined by micro Kjeldahls method Jackson (1973). N uptake by grain and straw of crop were calculated by multiplying dry matter production with corresponding values of their content and was expressed as kg ha^-1^.

### Available N in soil at harvest

Soil samples from each plot up to depth of 0–15 cm and 15–30 cm were taken for determination of available N in soil after harvest of the crop and were shade dried label wise on plain white papers for few days. The soil samples after drying were ground, sieved through 2 mm mesh and a composite sample was taken from each representative sample for further laboratory analysis.

### Nitrogen-use efficiencies

#### Agronomic efficiency (kg yield increase kg^-1^ nutrient applied)

Agronomic efficiency of nitrogen was computed by the formula given by Cassman and coworkers^39^ as

$$\mathrm{AE}=\frac{\left(\text{Grain yield in fertilized plot}-\text{Grain yield in control plot}\right) (\mathrm{kg})}{\text{Quantity of fertilizer applied }(\mathrm{kg})}$$

#### Partial factor productivity (kg grain kg^-1^ nutrient)

Partial factor productivity was calculated by the formula given by Cassman and coworkers^39^ as

$$\mathrm{PFP}=\frac{\text{Grain yield in fertilized plot }(\mathrm{kg})}{\text{Quantity of fertilizer applied }(\mathrm{kg})}$$

#### Apparent nutrient recovery (%)

Recovery efficiency of added nitrogen was computed by the formula given by Cassman and coworkers^39^ as

$$\text{RE }=\frac{\left(\text{Total nutrient uptake in fertilized plot}-\text{Total nutrient uptake in control plot}\right)(\mathrm{kg})}{\text{Quantity of fertilizer applied }(\mathrm{kg})} \times 100$$

#### Physiological efficiency (kg grain kg^-1^ nutrient)

Physiological efficiency of nitrogen was computed by the formula given by Baligar and coworkers^40^ as

$$\text{PE }=\frac{\left(\text{Grain yield in fertilized plot}-\text{Grain yield in control plot}\right)(\mathrm{kg})}{\left(\text{Total nutrient uptake in fertilized plot}-\text{Total nutrient uptake in control plot}\right)(\mathrm{kg})}$$

### Statistical analysis

Data on account of growth characteristics, yield contributing characters and yield was analysed using analysis of variance (ANOVA) at a 5% significance level for a two-factor split plot design, using OPSTAT software. The means were differentiated using a critical difference test at α = 0.05.

## Results

### N concentration and uptake by grain

Nitrogen concentration and uptake by rice grain were significantly influenced by both irrigation schedules and nitrogen levels (Table 3). Among irrigation treatments, recommended irrigation scheduling (I_1_) recorded the highest grain N concentration (1.023 and 1.012%) and uptake (88.02 and 85.39 kg ha⁻^1^) in 2021 and 2022, which was statistically similar to field capacity irrigation (I_2_) with N concentration of 1.015 and 0.989% and uptake of 84.07 and 81.03 kg ha⁻^1^. The lowest N concentration (0.960 and 0.935%) and uptake (50.35 and 47.36 kg ha⁻^1^) were observed under 20% depletion from field capacity (I_4_), indicating a substantial reduction compared to I_1_. Nitrogen levels also significantly affected grain N accumulation. Application of 125% RDN achieved the highest N concentration (1.018 and 1.005%) and uptake (82.21 and 79.73 kg ha⁻^1^), but these were statistically similar to 100% RDN, which recorded 1.003–0.982% N and 78.98–76.01 kg ha⁻^1^ uptake. The control treatment (N_0_) consistently showed the lowest grain N concentration and uptake. The interaction effect between irrigation schedules and nitrogen levels (I × N) was found to be non-significant during both years of experimentation.

**Table 3:** Influence of variable irrigation schedules and nitrogen levels on nitrogen content and uptake by grain and straw of rice.

Treatments	N content in grain (%)		N content in straw (%)		N uptake by grain (kg ha^-1^)		N uptake by straw (kg ha^-1^)		Total N uptake by crop (kg ha^-1^)	
	2021	2022	2021	2022	2021	2022	2021	2022	2021	2022
Irrigation schedules										
I_1_	1.023	1.012	0.528	0.502	88.02	85.39	58.06	57.97	146.08	143.36
I_2_	1.015	0.989	0.523	0.500	84.07	81.03	56.07	56.58	140.14	137.61
I_3_	0.970	0.943	0.504	0.474	62.55	59.07	44.34	44.78	106.89	103.85
I_4_	0.960	0.935	0.495	0.468	50.35	47.36	39.40	39.87	89.76	87.24
SE(m) ±	0.003	0.008	0.002	0.004	0.96	0.44	0.48	0.47	1.03	0.63
C.D. (p ≤ 0.05)	0.011	0.027	0.008	0.0013	3.42	1.55	1.72	1.66	3.64	2.42
Nitrogen levels										
N_0_	0.965	0.936	0.497	0.460	55.14	51.96	38.67	38.83	93.81	90.80
N_1_	0.983	0.955	0.508	0.483	68.66	65.15	47.60	47.97	116.27	113.13
N_2_	1.003	0.982	0.518	0.496	78.98	76.01	54.79	55.23	133.77	131.24
N_3_	1.018	1.005	0.528	0.505	82.21	79.73	56.81	57.17	139.02	136.91
SE(m) ±	0.003	0.004	0.003	0.005	0.63	0.77	0.39	0.54	0.72	1.07
C.D. (p ≤ 0.05)	0.009	0.013	0.009	0.015	1.85	2.26	1.15	1.60	2.12	3.16

I_1_: recommended irrigation scheduling, I_2_: at field capacity, I_3_: 10% depletion from field capacity, I_4_: 20% depletion from field capacity.

N_0_: Unfertilized control, N_1_: 75% RDN (recommended dose of nitrogen; @120 kg ha^-1^), N_2_: 100% RDN, N_3_: 125% RDN.

### N concentration and uptake by straw

The data pertaining to N concentration and uptake by straw of rice crop is presented in Table 3. Analysis of the data revealed that N concentration and uptake by straw of rice was significantly affected by irrigation schedules and nitrogen levels during both the years of experimentation.

Among the variable irrigation schedules, recommended irrigation scheduling (I_1_) recorded significantly highest N concentration and uptake by straw but was at par with the application of irrigation water at field capacity (I_2_) as compared to 10% depletion from field capacity (I_3_) and 20% depletion from field capacity (I_4_) treatments during both the years (2021 and 2022) of experimentation. The highest N concentration of 0.528 and 0.502 and uptake of 58.06 kg ha^-1^ and 57.97 kg ha^-1^ by straw was found in recommended irrigation scheduling followed by field capacity treatment which recorded the N concentration of 0.523 and 0.500 and uptake of 56.07 kg ha^-1^ and 56.58 kg ha^-1^ by straw at harvest during 2021 and 2022 respectively whereas the lowest N concentration of 0.495 and 0.468 and uptake of 39.40 kg ha^-1^ and 39.87 kg ha^-1^ by straw at harvest was recorded with the application of irrigation water at 20% depletion from field capacity respectively during both the years of experimentation.

Perusal of the data indicated that different nitrogen levels had significant effect on the N concentration and uptake by straw of rice during both the years. Among the different nitrogen levels, 125% RDN (recommended dose of nitrogen) recorded the highest N concentration and uptake by straw; however, it was at par with 100% RDN treatment at harvest of rice crop during both the years of experimentation. 125% RDN recorded significantly higher N concentration of 0.528 and 0.505 and uptake of 56.81 kg ha^-1^ and 57.17 kg ha^-1^ by straw at harvest followed by 100% RDN treatment which recorded the N concentration of 0.518 and 0.496 and uptake of 54.79 kg ha^-1^ and 55.23 kg ha^-1^ by straw during 2021 and 2022 respectively. Moreover, it was found that (N_0_) control treatment recorded significantly lowest N concentration and uptake by straw during both the years of experimentation. The interaction effect between irrigation schedules and nitrogen levels (I × N) was found to be non-significant during both years of experimentation.

### Total N uptake by rice crop

**Fig. 5:**
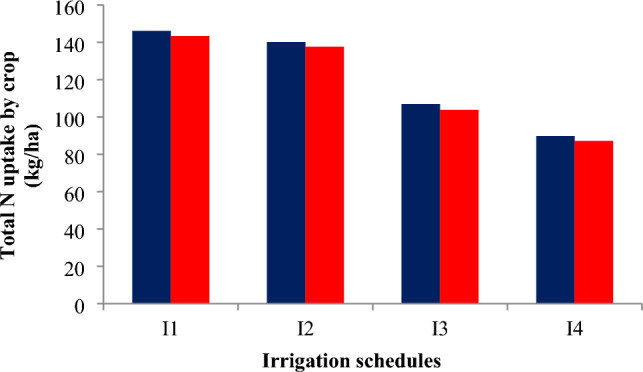
Total N uptake by crop under variable irrigation schedules during 2021 and 2022 crop growing seasons.

**Fig. 6:**
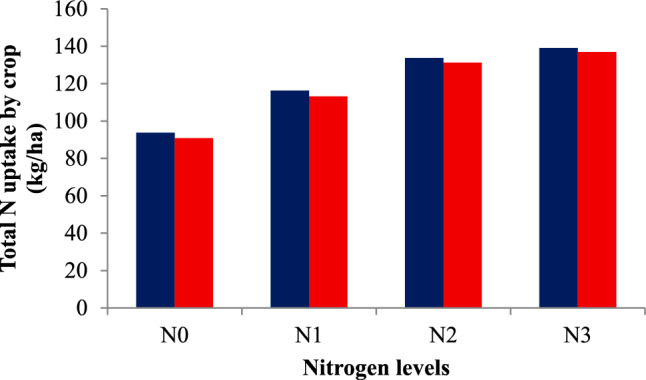
Total N uptake by crop under variable nitrogen levels during 2021 and 2022 crop growing seasons.

The total N uptake by rice was significantly influenced by both irrigation schedules and nitrogen levels (Table 3). Among irrigation treatments, recommended irrigation scheduling (I1) recorded the highest total N uptake of 146.08 and 143.36 kg ha⁻^1^ in 2021 and 2022, which was comparable to field capacity irrigation (I2) with 140.14 and 137.61 kg ha⁻^1^. Deficit irrigation at 20% depletion from field capacity (I4) reduced total N uptake by 38–39% compared to I1. Nitrogen levels also had a significant effect. Application of 125% RDN resulted in the highest total N uptake of 139.02 and 136.91 kg ha⁻^1^, but these values were statistically similar to 100% RDN, which recorded 133.77 and 131.24 kg ha⁻^1^ in 2021 and 2022, respectively. The control (N0) treatment consistently exhibited the lowest total N uptake, emphasizing the importance of adequate nitrogen fertilization for maximizing nutrient accumulation in rice (Figs. 5 and 6). The interaction effect between irrigation schedules and nitrogen levels (I × N) was found to be non-significant during both years of experimentation.

### Available N in soil at harvest

The data pertaining to available N in soil at harvest of rice crop at 0–15 cm and 15–30 cm soil depth is presented in Table 4. Analysis of the data revealed that available N in soil after harvest of rice crop was significantly affected by irrigation schedules and nitrogen levels during both the years of experimentation. Among the variable irrigation schedules, 20% depletion from field capacity treatment (I4) recorded significantly highest available N in soil (at 0–15 and 15–30 cm soil depth) at harvest as compared to 10% depletion from field capacity (I3) treatment, application of irrigation water at field capacity and recommended irrigation scheduling during both the years (2021 and 2022) of experimentation. It depicts that recommended irrigation scheduling removed more N from soil followed by application of irrigation water at field capacity and 10% and 20% depletion from field capacity. The highest available N in soil of 268.29 kg ha^-1^ and 261.39 kg ha^-1^ at 0–15 cm soil depth and 232.06 kg ha^-1^ and 222.84 kg ha^-1^ at 15–30 cm soil depth respectively was found in 20% depletion from field capacity treatment followed by application of irrigation water at 10% depletion from field capacity treatment which recorded the highest available N in soil of 251.75 kg ha^-1^ and 238.01 kg ha^-1^ at 0–15 cm soil depth and 223.28 kg ha^-1^ and 211.98 kg ha^-1^ at 15–30 cm soil depth during 2021 and 2022 respectively whereas the lowest available N in soil of 230.79 kg ha^-1^ and 214.55 kg ha^-1^ at 0–15 cm soil depth and 208.06 kg ha^-1^ and 190.09 kg ha^-1^ at 15–30 cm soil depth at harvest was recorded with the application of irrigation water at recommended irrigation scheduling respectively during both the years of experimentation.

**Table 4:** Influence of variable irrigation schedules and nitrogen levels on available nitrogen (kg ha^-1^) in soil at harvest of rice.

Treatments	Available nitrogen in soil at 0–15 cm soil depth (kg ha^-1^)		Available nitrogen in soil at 15–30 cm soil depth (kg ha^-1^)	
	2021	2022	2021	2022
Irrigation schedules				
I_1_	230.79	214.55	208.06	190.09
I_2_	244.35	230.93	219.01	198.42
I_3_	251.75	238.01	223.28	211.98
I_4_	268.29	261.39	232.06	222.84
SE(m) ±	1.65	4.16	2.43	2.46
C.D. (p ≤ 0.05)	5.82	14.70	8.58	8.69
Nitrogen levels				
N_0_	229.60	217.33	197.04	182.25
N_1_	244.56	231.35	211.24	196.57
N_2_	256.78	244.38	230.53	215.76
N_3_	264.24	251.81	243.60	228.75
SE(m) ±	3.55	2.80	3.06	3.07
C.D. (p ≤ 0.05)	10.42	8.22	14.99	14.01

I_1_: recommended irrigation scheduling, I_2_: at field capacity, I_3_: 10% depletion from field capacity, I_4_: 20% depletion from field capacity.

N_0_: Unfertilized control, N_1_: 75% RDN (recommended dose of nitrogen; @120 kg ha^-1^), N_2_: 100% RDN, N_3_: 125% RDN.

Perusal of the data indicated that different nitrogen levels had significant effect on the available N in soil at harvest of rice crop at 0–15 cm and 15–30 cm soil depth during both the years. Among the different nitrogen levels, 125% RDN (recommended dose of nitrogen) recorded the highest available N in soil at harvest of rice crop at 0–15 cm and 15–30 cm soil depth; however, it was at par with 100% RDN treatment at harvest of rice crop during both the years of experimentation. 125% RDN recorded significantly higher available N in soil at harvest of rice crop of 264.24 kg ha^-1^ and 251.81 kg ha^-1^ at 0–15 cm soil depth and 243.60 kg ha^-1^ and 228.75 kg ha^-1^ at 15–30 cm soil depth at harvest followed by 100% RDN treatment which recorded the available N in soil of 256.78 kg ha^-1^ and 244.38 kg ha^-1^ at 0–15 cm soil depth and 230.53 kg ha^-1^ and 215.76 kg ha^-1^ at 15–30 cm soil depth at harvest of rice crop during 2021 and 2022 respectively. Moreover, it was found that (N0) control treatment recorded significantly lowest available N in soil at harvest of rice crop at 0–15 cm and 15–30 cm soil depth during both the years of experimentation. The interaction effect between irrigation schedules and nitrogen levels (I × N) was found to be non-significant during both years of experimentation.

### Nitrogen use efficiencies

#### Agronomic efficiency (kg grain/kg N)

Agronomic efficiency of rice was significantly influenced by both irrigation schedules and nitrogen levels (Table 5). Among irrigation treatments, water application at field capacity (I_2_) recorded the highest agronomic efficiency (18.27 and 18.09 kg grain/kg N) in 2021 and 2022, which was statistically similar to recommended irrigation scheduling (I_1_) with 18.09 and 18.05 kg grain/kg N. The lowest efficiency (12.28 kg grain/kg N) was observed under 20% depletion from field capacity (I_4_), indicating reduced N utilization under deficit irrigation. Nitrogen levels also significantly affected agronomic efficiency. The 100% RDN treatment produced the highest efficiency (17.91 and 17.83 kg grain/kg N), followed by 125% RDN (15.62 kg grain/kg N) and 75% RDN, which recorded the lowest efficiency. Overall, irrigation at field capacity or recommended scheduling combined with 100% RDN optimized agronomic efficiency, whereas deficit irrigation or sub-optimal N levels reduced the crop’s ability to utilize applied nitrogen effectively.

**Table 5:** Influence of variable irrigation schedules and nitrogen levels on nitrogen use efficiencies of rice.

Treatments	Agronomic efficiency (kg grain increase/kg N applied)		Partial factor productivity (kg grain/kg N applied)		Apparent nutrient recovery (%)		Physiological efficiency (kg grain/kg N uptake)	
	2021	2022	2021	2022	2021	2022	2021	2022
Irrigation schedules								
I_1_	18.09	18.05	78.49	76.93	34.42	35.81	52.62	50.67
I_2_	18.27	18.09	75.79	74.66	35.19	35.40	51.79	51.52
I_3_	14.78	14.78	59.03	57.46	26.07	25.44	56.84	59.38
I_4_	12.28	12.28	48.32	46.75	22.17	22.33	55.43	55.15
SE(m) ±	1.15	1.15	0.93	0.73	1.44	2.56	0.79	0.75
C.D. (p ≤ 0.05)	3.05	3.08	3.29	2.57	5.10	6.04	2.42	2.34
Nitrogen levels								
N_0_	–	–	–	–	–	–	–	–
N_1_	14.04	13.95	77.30	75.42	24.95	24.81	56.50	57.20
N_2_	17.91	17.83	65.35	63.93	33.30	33.70	54.00	53.89
N_3_	15.62	15.62	53.57	52.50	30.14	30.74	52.01	51.44
SE(m) ±	0.56	0.63	0.56	0.64	0.53	0.90	0.69	0.66
C.D. (p ≤ 0.05)	1.70	1.92	1.71	1.94	1.60	2.72	2.11	1.99

I_1_: recommended irrigation scheduling, I_2_: at field capacity, I_3_: 10% depletion from field capacity, I_4_: 20% depletion from field capacity.

N_0_: Unfertilized control, N_1_: 75% RDN (recommended dose of nitrogen; @120 kg ha^-1^), N_2_: 100% RDN, N_3_: 125% RDN.

#### Partial factor productivity (kg grain/kg N applied)

The data pertaining to partial factor productivity is presented in Table 5. Analysis of the data revealed that partial factor productivity was significantly affected by irrigation schedules and nitrogen levels during both the years of experimentation. Among the variable irrigation schedules, recommended irrigation scheduling (I1) recorded significantly highest partial factor productivity but was at par with application of irrigation water at field capacity (I2) as compared to 10% depletion from field capacity (I3) and 20% depletion from field capacity (I4) treatments during both the years (2021 and 2022) of experimentation. The highest partial factor productivity of 18.27 kg grain/kg N and 18.09 kg grain/kg N was found in recommended irrigation scheduling but was at par with the application of irrigation water at field capacity which recorded the partial factor productivity of 18.09 kg grain/kg N and 18.05 kg grain/kg N at harvest during 2021 and 2022 respectively whereas the lowest partial factor productivity of 12.28 kg grain/kg N and 12.28 kg grain/kg N by rice was recorded with the application of irrigation water at 20% depletion from field capacity respectively during both the years of experimentation. Perusal of the data indicated that different nitrogen levels had significant effect on the partial factor productivity of rice during both the years. Among the different nitrogen levels, 75% RDN (recommended dose of nitrogen) recorded the highest partial factor productivity of rice followed by 100% RDN treatment and 125% RDN during both the years of experimentation. 75% RDN treatment recorded significantly higher partial factor productivity of 17.91 kg grain/kg N and 17.83 kg grain/kg N followed by 100% RDN treatment which recorded the partial factor productivity of 15.62 kg grain/kg N and 15.62 kg grain/kg N during 2021 and 2022 respectively. Moreover, it was found that 125% RDN treatment (N3) recorded significantly lowest partial factor productivity during both the years of experimentation.

#### Apparent nutrient recovery (%)

The data pertaining to apparent nutrient recovery is presented in Table 5. Analysis of the data revealed that apparent nutrient recovery was significantly affected by irrigation schedules and nitrogen levels during both the years of experimentation. Among the variable irrigation schedules, application of irrigation water at field capacity (I2) recorded significantly highest apparent nutrient recovery but was at par with recommended irrigation scheduling (I1) as compared to 10% depletion from field capacity (I3) and 20% depletion from field capacity (I4) treatments during both the years (2021 and 2022) of experimentation. The highest apparent nutrient recovery of 35.19% and 35.40% by rice was found in field capacity treatment followed by recommended irrigation scheduling which recorded the apparent nutrient recovery of 34.42 and 35.81% during 2021 and 2022 respectively whereas the lowest apparent nutrient recovery of 22.17 and 22.33% by rice was recorded with the application of irrigation water at 20% depletion from field capacity respectively during both the years of experimentation. Perusal of the data indicated that different nitrogen levels had significant effect on the apparent nutrient recovery of rice during both the years. Among the different nitrogen levels, 100% RDN (recommended dose of nitrogen) recorded the highest apparent nutrient recovery of rice followed by 125% RDN treatment and 75% RDN during both the years of experimentation. 100% RDN treatment recorded significantly higher apparent nutrient recovery of 33.30 and 33.70% followed by 125% RDN treatment which recorded the apparent nutrient recovery of 30.14% and 30.74% during 2021 and 2022 respectively. Moreover, it was found that 75% RDN treatment (N1) recorded significantly lowest apparent nutrient recovery during both the years of experimentation.

#### Physiological efficiency (kg grain/kg N uptake)

The data pertaining to physiological efficiency is presented in Table 5. Analysis of the data revealed that physiological efficiency was significantly affected by irrigation schedules and nitrogen levels during both the years of experimentation. Among the variable irrigation schedules, 10% depletion from field capacity (I3) recorded significantly highest physiological efficiency but was at par with 20% depletion from field capacity (I4) treatment as compared to recommended irrigation scheduling (I1) and application of irrigation water at field capacity (I2) during both the years (2021 and 2022) of experimentation. The highest physiological efficiency of 56.84 kg grain/kg N uptake and 59.38 kg grain/kg N uptake was found in 10% depletion from field capacity treatment but was at par with the application of irrigation water at 20% depletion from field capacity which recorded the physiological efficiency of 55.43 kg grain/kg N uptake and 55.15 kg grain/kg N uptake during 2021 and 2022 respectively whereas the lowest physiological efficiency of 51.79 kg grain/kg N uptake and 51.52 kg grain/kg N uptake by rice was recorded with the application of irrigation water at field capacity respectively during both the years of experimentation. Perusal of the data indicated that different nitrogen levels had significant effect on the physiological efficiency of rice during both the years. Among the different nitrogen levels, 75% RDN (recommended dose of nitrogen) recorded the highest physiological efficiency of rice followed by 100% RDN treatment and 125% RDN during both the years of experimentation. 75% RDN treatment recorded significantly higher physiological efficiency of 56.50 kg grain/kg N uptake and 57.20 kg grain/kg N uptake followed by 100% RDN treatment which recorded the physiological efficiency of 54 kg grain/kg N uptake and 53.89 kg grain/kg N uptake during 2021 and 2022 respectively. Moreover, it was found that 125% RDN treatment (N_1_) recorded significantly lowest physiological efficiency during both the years of experimentation. The interaction effect between irrigation schedules and nitrogen levels (I × N) was found to be non-significant for nitrogen use efficiencies during both years of experimentation.

### Water saving (%) and yield loss (%) in rice under variable irrigation schedules

The data pertaining to number of irrigations required, amount of irrigation water required, water saving and yield loss is presented in Table 6. It was revealed that irrigation regimes affected the number of irrigations required, amount of irrigation water required, water saving and yield loss of rice during both the years of experimentation. Among the variable irrigation schedules, recommended irrigation scheduling (I_1_) treatment required highest no. of irrigations (43 and 40) and highest amount of irrigation water (2150 mm and 2000 mm), followed by application of irrigation water at field capacity (I_2_) which required 33 and 34 number of irrigations and 660 mm and 680 mm amount of irrigation water during 2021 and 2022 respectively. The lowest number of irrigations (14 and 15) and amount of irrigation water (280 mm and 300 mm) was required in application of irrigation water at 20% depletion from field capacity during both the years of experimentation. Perusal of the data indicated that different irrigation schedules had significant effect on the water saving and yield loss of rice during both the years. Among the different irrigation regimes, application of irrigation water at 20% depletion from field capacity (I_4_) recorded highest water saving (86.97 and 85%) and yield loss (39.04 and 39.88%), followed by 10% depletion from field capacity treatment which recorded 83.25 and 81% saving of irrigation water but with a yield loss of 25.05 and 25.59% respectively over recommended irrigation scheduling during both the years of experimentation. Application of irrigation water at field capacity recorded the water saving of 69.30% and 66% with a yield loss of 3.61% and 2.97% over recommended irrigation scheduling treatment during 2021 and 2022 respectively.

**Table 6:** Water saving (%) and yield loss (%) in rice under variable irrigation schedules.

Treatments	No. of irrigations		Irrigation water applied (mm)		Water saving (%)		Yield loss (%)	
	2021	2022	2021	2022	2021	2022	2021	2022
Irrigation schedules								
I_1-_Recommended irrigation scheduling	43	40	2150	2000	–	–	–	–
I_2_-At field capacity	33	34	660	680	69.30	66.00	3.61	2.97
I_3_-10% depletion from field capacity	18	19	360	380	83.25	81.00	25.05	25.59
I_4_-20% depletion from field capacity	14	15	280	300	86.97	85.00	39.04	39.88

### Crop water productivity

The data pertaining to crop water productivity is presented in Table 7. Analysis of the data revealed that crop water productivity was significantly affected by irrigation schedules and nitrogen levels during both the years of experimentation. Among the variable irrigation schedules, application of irrigation water at 10% depletion from field capacity (I_3_) recorded significantly highest crop water productivity but was at par with 20% depletion from field capacity treatment (I_4_) as compared to field capacity (I_2_) and recommended irrigation scheduling (I_1_) treatments during both the years (2021 and 2022) of experimentation. The highest crop water productivity of 1.30 kg grain m^-3^ and 1.53 kg grain m^-3^ was found in application of irrigation water at 10% depletion from field capacity treatment followed by 20% depletion from field capacity treatment which recorded the crop water productivity of 1.25 kg grain m^-3^ and 1.46 kg grain m^-3^ during 2021 and 2022 respectively whereas the lowest crop water productivity of 1.06 kg grain m^-3^ and 1.07 kg grain m^-3^ was recorded with the application of irrigation water at recommended irrigation scheduling respectively during both the years of experimentation. Perusal of the data indicated that different nitrogen levels had significant effect on the crop water productivity of rice during both the years. Among the different nitrogen levels, 125% RDN (recommended dose of nitrogen) recorded the highest crop water productivity but was at par with 100% RDN treatment as compared to 75% RDN and control treatment during both the years of experimentation. 125% RDN treatment recorded significantly higher crop water productivity of 1.33 kg grain m^-3^ and 1.47 kg grain m^-3^ followed by 100% RDN treatment which recorded the crop water productivity of 1.30 kg grain m^-3^ and 1.47 kg grain m^-3^ during 2021 and 2022 respectively. Moreover, it was found that control treatment (N_0_) recorded significantly lowest crop water productivity during both the years of experimentation.

**Table 7:** Influence of variable irrigation schedules and nitrogen levels on crop water productivity (kg m^-3^) of rice.

Treatments	Crop water productivity (kg grain m^-3^)	
	2021	2022
Irrigation schedules		
I_1_	1.06	1.07
I_2_	1.12	1.15
I_3_	1.30	1.53
I_4_	1.25	1.46
SE(m) ±	0.01	0.02
C.D. (p ≤ 0.05)	0.05	0.06
Nitrogen levels		
N_0_	0.94	1.03
N_1_	1.16	1.27
N_2_	1.30	1.43
N_3_	1.33	1.47
SE(m) ±	0.01	0.02
C.D. (p ≤ 0.05)	0.03	0.5

I_1_: recommended irrigation scheduling, I_2_: at field capacity, I_3_: 10% depletion from field capacity, I_4_: 20% depletion from field capacity.

N_0_: Unfertilized control, N_1_: 75% RDN (recommended dose of nitrogen; @120 kg ha^-1^), N_2_: 100% RDN, N_3_: 125% RDN.

### Relative economics

The data on total cost of cultivation, gross returns, net returns, and benefit–cost (B:C) ratio are presented in Table 8. Relative economics were calculated considering the cost of production for each treatment and the corresponding marketable yield at prevailing prices. The pooled economic analysis for 2021 and 2022 indicate that the highest net returns were obtained with irrigation at field capacity combined with nitrogen application at 125% of RDN (I2N3). This treatment also recorded the highest B:C ratio of 1.64. In contrast, the lowest net returns and B:C ratio (0.30) were observed under irrigation at 20% depletion from field capacity without nitrogen application (control).

**Table 8:** Economics of different treatments (pooled over 2021 and 2022).

Treatment combinations	Total cost of cultivation (₹)	Gross returns from grain yield (₹)	Gross returns from straw yield (₹)	Total returns (₹)	Net returns (₹)	BC ratio
I_1_N_0_	64,300	89,052.17	28,882.5	117,934.7	53,634.7	0.83
I_1_N_1_	65,170	107,076.7	33,635	140,711.7	75,541.7	1.16
I_1_N_2_	65,560	121,721.2	38,740	160,461.2	94,901.2	1.45
I_1_N_3_	65,950	124,017.8	39,355	163,372.8	97,422.8	1.48
I_2_N_0_	58,680	85,245.33	28,229	113,474.3	54,794.3	0.93
I_2_N_1_	59,550	103,406.3	33,470	136,876.3	77,326.3	1.30
I_2_N_2_	59,940	117,834.2	38,265	156,099.2	96,159.2	1.60
I_2_N_3_	60,330	120,477.5	38,850	159,327.5	98,997.5	1.64
I_3_N_0_	57,480	64,915.5	23,942.5	88,858	31,378.0	0.55
I_3_N_1_	58,350	79,302.17	28,944	108,246.2	49,896.2	0.86
I_3_N_2_	58,740	91,433.33	30,806	122,239.3	63,499.3	1.08
I_3_N_3_	59,130	94,293.33	31,258.5	125,551.8	66,421.8	1.12
I_4_N_0_	57,160	52,652.17	21,601	74,253.17	17,093.2	0.30
I_4_N_1_	58,030	67,600	25,689	93,289	35,259.0	0.61
I_4_N_2_	58,420	72,407.83	28,322	100,729.8	42,309.8	0.72
I_4_N_3_	58,810	74,925.5	28,862	103,787.5	44,977.5	0.76

Details of treatments:

I_1_ = Recommended irrigation scheduling, I_2_ = At field capacity, I_3_ = 10% depletion from field capacity, I_4_ = 20% depletion from field capacity.

N_0_ = Control, N_1_ = 75% Recommended dose of nitrogen (RDN), N_2_ = 100% RDN, N_3_ = 125% RDN.

## Discussion

### Post-harvest plant and soil nutrient studies

#### N concentration and uptake by grain and straw of crop

Nitrogen concentration and uptake by rice grain and straw were significantly influenced by irrigation schedules and nitrogen levels during both years. Recommended irrigation scheduling recorded the highest N concentration and uptake, closely followed by irrigation at field capacity, whereas deficit irrigation at 10% and 20% depletion from field capacity resulted in lower values. The higher N content and uptake under sufficient soil moisture conditions may be attributed to improved soil–plant water relations, enhanced root growth, and better translocation of nitrogen from roots to shoots and grains. Adequate moisture and optimal temperature also favour microbial activity, leading to improved mineralization and nitrification, thus increasing nitrogen availability in the rhizosphere. Conversely, soil drying under deficit irrigation likely restricted microbial and enzymatic activities, reduced root proliferation with constrained microbial mineralization resulting in limited N availability and uptake. These findings are consistent with those of Chowdhury et al.^41^ and Wang et al.^42^. Among nitrogen levels, 125% RDN produced the highest N concentration and uptake, statistically similar to 100% RDN, while the control showed the lowest values. The enhanced N content and uptake at higher N rates can be attributed to increased nitrogen availability, leading to improved vegetative growth, leaf area expansion, and photosynthetic efficiency. This enhanced biomass accumulation and tiller production ultimately resulted in higher nitrogen uptake by grain and straw, in agreement with Sandhu and Mahal^43^ and Elina et al.^44^.

#### Available N in soil at harvest

Available soil nitrogen after harvest was significantly affected by both irrigation schedules and nitrogen levels. The 20% depletion from field capacity treatment recorded the highest available soil N at both 0–15 cm and 15–30 cm depths, indicating reduced N removal by plants under deficit moisture conditions. In contrast, recommended irrigation scheduling and field capacity treatments showed the lowest available soil N due to higher plant uptake and possible N losses via denitrification and leaching under wet conditions. The higher available N in deficit irrigation treatments may also reflect immobilization of N and reduced mineralization due to limited microbial activity under moisture stress. These interpretations are supported by the findings of Amgain et al.^45^ and Wang et al.^46^.

Similarly, higher nitrogen application rates (125% and 100% RDN) maintained higher residual soil N compared to lower N levels, indicating that excess N supply beyond plant demand results in accumulation in soil. Increased microbial activity and faster mineralization at higher N doses may also contribute to greater available soil N after harvest (Malav et al.^47^ and Kabat and Satapathy^48^).

### Nitrogen use efficiencies

#### Agronomic efficiency

Agronomic nitrogen use efficiency (ANUE) was significantly influenced by both irrigation and nitrogen treatments. Irrigation at field capacity resulted in the highest ANUE, statistically at par with recommended irrigation scheduling. Optimal soil moisture likely improved root aeration and oxygen availability, promoting root health and nutrient absorption. Furthermore, balanced water conditions facilitated favourable biochemical processes such as nitrification and mineralization, leading to improved nitrogen availability and uptake efficiency. Excess or deficit irrigation disturbed water–air balance, resulting in nitrogen losses or reduced uptake efficiency. These results align with those reported by Mboyerwa et al.^49^ and Thakur et al.^50^.

Among nitrogen levels, 100% RDN exhibited the highest ANUE, which declined at higher nitrogen levels (125% RDN). Excessive N application likely increased unproductive tillers and vegetative growth, reducing nitrogen conversion efficiency into grain yield. Similar trends were reported by Zhang et al.^51^ and Wang et al.^42^.

#### Partial factor productivity

The results revealed that partial factor productivity was significantly affected by irrigation schedules and nitrogen levels during both the years of experimentation. Among the variable irrigation schedules, recommended irrigation scheduling recorded significantly higher partial factor productivity but was at par with application of irrigation water at field capacity. Under sufficient soil moisture condition, there is improvement in access to water and nutrient in the top soil layer resulting in higher grain filling rates in rice, thus offers the opportunity to significantly improve rice PFPN. This is in close conformity with the findings of Yang et al*.*^52^. Among the different nitrogen levels, 75% RDN (recommended dose of nitrogen) recorded the highest partial factor productivity of rice followed by 100% RDN treatment and 125% RDN during both the years of experimentation. It was found that 125% RDN treatment recorded significantly lower partial factor productivity during both the years of experimentation. At higher nitrogen levels partial factor productivity (PFPN) of rice decreased due to the fact that increased nitrogen application increased the number of unproductive tillers and more vegetative growth leading to low PFP. The results are in line with the findings of Sharma et al*.*^53^.

#### Apparent nutrient recovery

The data revealed that apparent nutrient recovery was significantly affected by irrigation schedules and nitrogen levels during both the years of experimentation. Among the variable irrigation schedules, application of irrigation water at field capacity recorded significantly higher apparent nutrient recovery but was at par with recommended irrigation scheduling during both the years. The lower apparent nutrient recovery (REN) values in flooded rice (recommended irrigation scheduling) are best possible due to greater N losses through leaching, denitrification and ammonia volatilization. Similar results have been reported by Gupta et al*.*^54^. Among the different nitrogen levels, 100% RDN (recommended dose of nitrogen) recorded the highest apparent nutrient recovery of rice followed by 125% RDN treatment and 75% RDN during both the years of experimentation. At higher N application, apparent nutrient recovery decreased due to the fact that nitrogen was lost in the form of leaching, denitrification and ammonia volatilization. The results are in line with the findings of Gupta et al*.*^54^.

#### Physiological efficiency

The results revealed that physiological efficiency was significantly affected by irrigation schedules and nitrogen levels during both the years of experimentation. Among the variable irrigation schedules, 10% depletion from field capacity recorded significantly higher physiological efficiency but was at par with 20% depletion from field capacity treatment as compared to recommended irrigation scheduling and application of irrigation water at field capacity during both the years of experimentation. The results are in line with the findings of Kour et al*.*^55^ and Singh et al*.*^56^. Among the different nitrogen levels, 75% RDN (recommended dose of nitrogen) recorded the highest physiological efficiency of rice followed by 100% RDN treatment and 125% RDN during both the years of experimentation. 125% RDN treatment recorded significantly lowest physiological efficiency during both the years of experimentation. The results are in close conformity with the findings of Hazra and Chandra^57^, Liu et al*.*^58^ and Mir et al*.*^59^.

#### Crop water productivity

The results revealed that crop water productivity was significantly affected by irrigation schedules and nitrogen levels during both years of experimentation. Among the variable irrigation schedules, application of irrigation water at 10% depletion from field capacity recorded the highest crop water productivity but was at par with 20% depletion from field capacity treatment as compared to field capacity and recommended irrigation scheduling treatments during both the years of experimentation. This may be attributed to the fact that deficit irrigation reduced the amount of water loss through evaporation, seepage, and percolation since water was not always kept above the soil surface and consequently reduced water use and increased the percentage of water saved when compared to the continuous submergence treatment. This outcome agrees with Chu et al.^60^ and Liu et al.^61^. More irrigation input enhanced the rice yield but reduced the water use efficiency. The results are supported by Liang et al.^62^. Among the different nitrogen levels, 125% RDN recorded the highest crop water productivity but was at par with 100% RDN treatment. Control treatment recorded the lowest crop water productivity during both years of experimentation. Increased crop water productivity was the result of higher rice yields under increased N application rates. This is in line with the findings of Ren et al.^63^ and Zanini et al.^64^.

## Conclusion

The study demonstrated that irrigation scheduling and nitrogen management jointly regulate nitrogen dynamics, uptake, and use efficiency in rice under temperate conditions. Optimal soil moisture (field capacity or recommended irrigation) enhanced nitrogen uptake and translocation, while deficit irrigation improved physiological efficiency but reduced total uptake. Nitrogen at 100% RDN offered the best balance between yield and nitrogen efficiency, whereas 125% RDN led to nutrient accumulation and reduced use efficiency. These findings underline the importance of synchronizing irrigation with crop water demand and aligning nitrogen supply with plant requirement to optimize nutrient use, reduce environmental losses, and enhance sustainable rice productivity in temperate Himalayan ecosystems.

## Supplementary Information

Supplementary Information.

## Data Availability

The datasets used and/or analysed during the current study available from the corresponding author on reasonable request.
