# Supplementary material for: Optimizing irrigation and nitrogen levels for improved soil nitrogen dynamics and use efficiency in temperate ecology of Kashmir
Source: Sci Rep. 2025 Dec 30;16:2722. doi: 10.1038/s41598-025-32465-1 (PMC12823565; doi:10.1038/s41598-025-32465-1)
Supplement: Supplementary file 1 — Supplementary Information. [file 41598_2025_32465_MOESM1_ESM.docx]

**Supplementary Table 1: Soil water balance under variable irrigation schedules and nitrogen levels (Year 2021)**

| **Treatments** | **Irrigation (mm)** | **Rainfall (mm)** | **Total input (I+R)** | **ETc**  **(mm)** | **Ds**  **(mm)** | **Total**  **(ETc + Ds)** | **Deep percolation (mm)** | **Total output** | **Balance** |
| --- | --- | --- | --- | --- | --- | --- | --- | --- | --- |
| **I_1_N_0_** | 2150 | 419.5 | 2569.5 | 804.3 | 54 | 858.3 | 1711.2 | 2569.5 | 0 |
| **I_1_N_1_** | 2150 | 419.5 | 2569.5 | 804.3 | 54 | 858.3 | 1711.2 | 2569.5 | 0 |
| **I_1_N_2_** | 2150 | 419.5 | 2569.5 | 804.3 | 54 | 858.3 | 1711.2 | 2569.5 | 0 |
| **I_1_N_3_** | 2150 | 419.5 | 2569.5 | 804.3 | 54 | 858.3 | 1711.2 | 2569.5 | 0 |
| **I_2_N_0_** | 660 | 419.5 | 1079.5 | 736.9 | 29 | 765.9 | 313.6 | 1079.5 | 0 |
| **I_2_N_1_** | 660 | 419.5 | 1079.5 | 736.9 | 29 | 765.9 | 313.6 | 1079.5 | 0 |
| **I_2_N_2_** | 660 | 419.5 | 1079.5 | 736.9 | 29 | 765.9 | 313.6 | 1079.5 | 0 |
| **I_2_N_3_** | 660 | 419.5 | 1079.5 | 736.9 | 29 | 765.9 | 313.6 | 1079.5 | 0 |
| **I_3_N_0_** | 360 | 419.5 | 779.5 | 493.9 | 35 | 528.9 | 250.6 | 779.5 | 0 |
| **I_3_N_1_** | 360 | 419.5 | 779.5 | 493.9 | 35 | 528.9 | 250.6 | 779.5 | 0 |
| **I_3_N_2_** | 360 | 419.5 | 779.5 | 493.9 | 35 | 528.9 | 250.6 | 779.5 | 0 |
| **I_3_N_3_** | 360 | 419.5 | 779.5 | 493.9 | 35 | 528.9 | 250.6 | 779.5 | 0 |
| **I_4_N_0_** | 280 | 419.5 | 699.5 | 415.8 | 31 | 446.8 | 252.7 | 699.5 | 0 |
| **I_4_N_1_** | 280 | 419.5 | 699.5 | 415.8 | 31 | 446.8 | 252.7 | 699.5 | 0 |
| **I_4_N_2_** | 280 | 419.5 | 699.5 | 415.8 | 31 | 446.8 | 252.7 | 699.5 | 0 |
| **I_4_N_3_** | 280 | 419.5 | 699.5 | 415.8 | 31 | 446.8 | 252.7 | 699.5 | 0 |

**Supplementary Table 2: Soil water balance under variable irrigation schedules and nitrogen levels (Year 2022)**

| **Treatments** | **Irrigation (mm)** | **Rainfall (mm)** | **Total input (I+R)** | **ETc**  **(mm)** | **Ds**  **(mm)** | **Total**  **(ETc + Ds)** | **Deep percolation (mm)** | **Total output** | **Balance** |
| --- | --- | --- | --- | --- | --- | --- | --- | --- | --- |
| **I_1_N_0_** | 2000 | 384.4 | 2384.4 | 783.4 | 41 | 824.4 | 1560 | 2384.4 | 0 |
| **I_1_N_1_** | 2000 | 384.4 | 2384.4 | 783.4 | 41 | 824.4 | 1560 | 2384.4 | 0 |
| **I_1_N_2_** | 2000 | 384.4 | 2384.4 | 783.4 | 41 | 824.4 | 1560 | 2384.4 | 0 |
| **I_1_N_3_** | 2000 | 384.4 | 2384.4 | 783.4 | 41 | 824.4 | 1560 | 2384.4 | 0 |
| **I_2_N_0_** | 680 | 384.4 | 1064.4 | 706.8 | 30 | 736.8 | 327.6 | 1064.4 | 0 |
| **I_2_N_1_** | 680 | 384.4 | 1064.4 | 706.8 | 30 | 736.8 | 327.6 | 1064.4 | 0 |
| **I_2_N_2_** | 680 | 384.4 | 1064.4 | 706.8 | 30 | 736.8 | 327.6 | 1064.4 | 0 |
| **I_2_N_3_** | 680 | 384.4 | 1064.4 | 706.8 | 30 | 736.8 | 327.6 | 1064.4 | 0 |
| **I_3_N_0_** | 380 | 384.4 | 764.4 | 406.5 | 26 | 432.5 | 331.9 | 764.4 | 0 |
| **I_3_N_1_** | 380 | 384.4 | 764.4 | 406.5 | 26 | 432.5 | 331.9 | 764.4 | 0 |
| **I_3_N_2_** | 380 | 384.4 | 764.4 | 406.5 | 26 | 432.5 | 331.9 | 764.4 | 0 |
| **I_3_N_3_** | 380 | 384.4 | 764.4 | 406.5 | 26 | 432.5 | 331.9 | 764.4 | 0 |
| **I_4_N_0_** | 300 | 384.4 | 684.4 | 346.3 | 41 | 387.3 | 297.1 | 684.4 | 0 |
| **I_4_N_1_** | 300 | 384.4 | 684.4 | 346.3 | 41 | 387.3 | 297.1 | 684.4 | 0 |
| **I_4_N_2_** | 300 | 384.4 | 684.4 | 346.3 | 41 | 387.3 | 297.1 | 684.4 | 0 |
| **I_4_N_3_** | 300 | 384.4 | 684.4 | 346.3 | 41 | 387.3 | 297.1 | 684.4 | 0 |
